# Endometriosis Predictive Models Based on Self-Assessment Questionnaire, Evidence from Clinical Examination or Imaging Findings: A Narrative Review

**DOI:** 10.3390/jcm13020356

**Published:** 2024-01-08

**Authors:** Fani Gkrozou, Orestis Tsonis, Felice Sorrentino, Luigi Nappi, Anastasia Vatopoulou, Chara Skentou, Suruchi Pandey, Minas Paschopoulos, Angelos Daniilidis

**Affiliations:** 1 Department of Obstetrics and Gynaecology, University Hospital of Ioannina, 451 10 Ioannina, Greece; fani.gkrozou@gmail.com (F.G.); haraskentou@gmail.com (C.S.); mpaschop@gmail.com (M.P.); 2 Assisted Conception Unit, Guy’s and St Thomas’ Hospital NHS Foundation Trust, London SE1 9RT, UK; orestis.tsonis@gmail.com; 3 Department of Gynaecology, St George’s University Hospitals NHS Foundation Trust, London WC1E 6BT, UK; ange1972@otenet.gr; 4 Department of Medical and Surgical Sciences, Institute of Obstetrics and Gynecology, University of Foggia, 71121 Foggia, Italy; luigi.nappi@unifg.it; 5 2nd Department of Obstetrics and Gynaecology, Hippokration General Hospital, Aristotle University of Thessaloniki, 541 24 Thessaloniki, Greece

**Keywords:** endometriosis, predictive models, self-assessment questionnaire, chronic pelvic pain, treatment, endometriosis, pregnancy

## Abstract

Objective: The aim of this narrative review is to evaluate existing questionnaires on predictive models for endometriosis. These symptom-based models have the potential to serve as screening tools for adult women to detect endometriosis. Data sources: A comprehensive search of PubMed and Embase databases was conducted to identify studies on endometriosis screening. Selection of studies: The search targeted predictive models for endometriosis localisation, bowel involvement, need for bowel surgery and fertility. Due to the heterogeneity identified, a systematic review was not possible. A total of 23 studies were identified. Data extraction and synthesis: Among these studies, twelve included measures for general endometriosis, two targeted specific sites, four focused on deep infiltrating endometriosis (DIE), and three addressed the need for endometriosis-related bowel surgery. Many measures combined clinical, imaging and laboratory tests with patient questionnaires. Validation of these models as screening tools was lacking in all studies, as the focus was on diagnosis rather than screening. Conclusion: This review did not identify any fully validated, symptom-based questionnaires for endometriosis screening in adult women. Substantial validation work remains to establish the efficacy of such tools.

## 1. Introduction

Endometriosis is a persistent, estrogen-related disease characterised by the presence of endometrial-like tissue outside the uterus. This abnormality triggers inflammatory reactions and scarring of the tissue [1]. The exact prevalence of endometriosis remains difficult to determine, although it is estimated that approximately 10% of reproductive-age females have endometriosis with 30–50% of them experiencing pelvic pain and/or infertility. Especially in regions with higher socio-demographic indices, the prevalence and incidence of the disease increased significantly between 1990 and 2017 [2]. The consequences of endometriosis include chronic pelvic pain, dyspareunia, infertility and a reduced quality of life (QoL) [1]. In women who struggle with chronic pelvic pain due to endometriosis, the impairment of quality of life is more pronounced than in women with pelvic pain due to other causes. This chronic condition primarily affects areas such as pain, stress, anxiety and social adjustment. In addition, research has looked at the impact of endometriosis on everyday activities such as work, social bonding, sexuality and psychological wellbeing. The symptoms experienced by people with endometriosis cover a broad spectrum. In particular, deep infiltrating endometriosis (DIE), in which endometrial-like tissue extends at least 5 mm beyond the lining of the uterus, accounts for almost 20% of endometriosis cases [3]. However, even today, diagnosing endometriosis and determining the factors that influence its progression and associated symptoms is still a huge challenge. The process of diagnosing endometriosis often takes years, which adds to the frustration and anxiety of patients. As a result, treatment and care for these women is delayed, sometimes up to 6–12 years after the first onset of symptoms [4]. The available evidence favours active patient involvement in the detection and diagnosis of the disease. However, relying on pain alone is not enough to diagnose endometriosis. Apart from the complexity of the diagnosis and the prognosis of symptoms, there is also the question of the proportion of women who experience improvement after surgery [5]. Another pressing concern is the prediction of fertility problems in women with endometriosis. It is clear that endometriosis profoundly affects various facets of women’s lives. Consequently, the development of predictive models is essential to identify risk factors for the development of endometriosis, the symptoms and the severity of the disease. The aim of this study is to present predictive models from the existing literature based on self-assessment questionnaires with or without imaging examinations. These questionnaires are focused on women’s symptoms, life style, family history and the effect of pain at their everyday life. The central questions of this study include the prediction of early-stage endometriosis or deep infiltrating endometriosis (DIE), the preoperative localisation of endometriosis, and particularly the identification of bowel involvement. The development of efficient predictive models will help primary care physicians to identify and diagnose women with endometriosis and improve the service they offer. As a result, these patients will be treated earlier and potentially better by specialists. Specialists will be able to recognise and offer the best treatment according to the models and improve women’s life. Although some predictive models have emerged in the literature, such as anti-Mullerian hormone or urinary peptide patterns, these efforts are preliminary and lack the extensive data required for clinical integration [6]. The diversity of studies further complicates statistical synthesis and analysis. Furthermore, the heterogeneity within the endometriosis population exacerbates the challenges faced in subsequent analyses.

## 2. Materials and Methods

For this study, we conducted an extensive narrative literature search covering the years 2005 to 2022. We meticulously searched databases such as PubMed and EMBASE using strategic keywords such as “endometriosis AND predictive models”, “fertility AND endometriosis AND predictive models” and “bowel surgery for endometriosis AND predictive models” (Table 1).

Our selection criteria included studies with patient-completed questionnaires and symptom-based screening tools. In addition, we included studies that looked at models that considered the localisation of endometriosis using imaging both in conjunction with questionnaires and independently. However, to maintain accuracy and focus, we deliberately excluded models that focused exclusively on postoperative findings or studies that focused exclusively on adolescent cohorts (Table 2).

From the original pool of 180 articles, 52 were identified as duplicates, 128 were excluded based on predetermined criteria, and a curated selection of 23 studies were found to fit our research objectives. To increase rigour, the studies that were deemed appropriate were reviewed by two different and impartial researchers. The selected studies were characterised by a great diversity, differing in design, objectives and results. Due to this heterogeneity, it was not possible to provide a comprehensive systematic review of the prediction models presented. Instead, Table 3 provides essential insights into the key features of each included study, explaining the population studied, the geographical context, the type of analytical tool used, a brief overview of the methodology and the clinical significance of the results. It is important to note that this narrative review is an attempt to distil and consolidate the wealth of information available given the complex landscape of predictive models associated with endometriosis.

## 3. Predictive Models of Endometriosis at Early Stages

Given the noticeable delay in endometriosis diagnosis, which affects women’s well-being and has a potential impact on fertility [17,29], Verket et al. ventured to create a prediction model to facilitate early identification of women at high risk of endometriosis in primary care [8]. Their study relied on an anonymous questionnaire that revealed a robust association between a family history of endometriosis and future manifestation of the disease [8]. Parallel studies focused on pain as a predictive factor [14,17], but its subjective nature limited its prognostic utility. Of note, the risk of endometriosis was almost 50% higher in women with a family history of endometriosis [21,22], emphasizing the crucial role of this factor in early detection [30]. In other endeavours, Forman et al. [9] developed a questionnaire that focused on women’s pain and health history to differentiate those with endometriosis from those with a healthy pelvis. Unfortunately, this questionnaire did not effectively differentiate between the two groups. Fasciani et al. [11] proposed an endometriosis index composed of 38 parameters, taking into account patient-reported pain, consultation with physicians and diagnostic evidence, including pelvic examinations, imaging and laboratory tests. In contrast, Yeung et al. [12] developed a mathematical model using a preoperative questionnaire similar to the World Endometriosis Research Foundation-Women’s Health Symptom Survey (WERF-WHSS). Although the model had a sensitivity of 80.5% and a specificity of 57.7%, it was difficult for clinical application due to its complicated nature. Five studies that take a different perspective examine pre-surgical prediction models [8,14]. The approach of Eskenazi et al. [13] aimed to predict surgical diagnoses based on patient interviews, combining clinical symptoms, history, examinations and ultrasound findings. Although this model was excellent in predicting ovarian endometriosis, its efficacy diminished for non-ovarian manifestations. Calhaz-Jorge et al. [14] attempted to predict endometriosis in sub fertile women using a questionnaire that included age at laparoscopy, menarche, demographic data, social variables, obstetric history and cycle characteristics. This study, although informative, was not validated beyond its sub fertile study population and included women with previous pelvic surgery [14]. The study by Ballard et al. [15] aimed to determine whether endometriosis cases could be recognised prior to laparoscopy based on pain characteristics, using a comprehensive questionnaire to assess various aspects of pain. Dyschezia was found to be more common in women with endometriosis, particularly those with deep infiltrative endometriosis. While this study is informative, it has yet to be validated. In the meantime, Hackethal et al. [16] examined medical records to formulate a comprehensive questionnaire covering endometriosis history, surgical history, allergies, chronic diseases, family history, fertility and obstetric history. Although the questionnaire is rich in data, its complexity compromises the usefulness of self-assessment, and the differential diagnosis of women with and without endometriosis was not prioritized [16]. An innovative study in 2012 introduced a symptom-based model that predicted not only the presence of endometriosis but also its different stages in symptomatic women without prior surgical diagnosis [31]. While the prediction for each endometriosis stage remained modest, the accuracy improved when ultrasound findings of ovarian endometriotic cysts or nodules were taken into account. The prediction for stage III/IV endometriosis in particular achieved a high level of accuracy. With the Endometriosis Research Centre’s self-test, a questionnaire was introduced that allowed women to self-assess the likelihood of endometriosis based on their symptoms and medical history [18]. Respondents who answered “yes” to three or more questions showed an increased potential for endometriosis, even in non-symptomatic areas such as family history, subfertility or miscarriage. Abdulai et al. [7] took a digital approach and developed a web-based system for women with endometriosis that serves as both an educational tool and a platform for self-assessment. However, the user-friendliness of the system proved to be insufficient, so that there was no significant user participation.

## 4. Predictive Models for Deep Infiltrating Endometriosis

In the field of predictive modelling for deep infiltrating endometriosis (DIE), Chapron et al. [21] have developed an innovative approach using a self-assessment questionnaire to predict the presence of DIE in women struggling with chronic pelvic pain. In this ground-breaking study, a range of prevalent symptoms such as dysmenorrhoea, dyspareunia and bowel and urinary symptoms during menstruation were carefully recorded. It is noteworthy that posterior DIE was closely associated with the leading symptom of dyschezia. In contrast to the conventional questionnaires filled out by patients, this study was driven by the development of a complex predictive model. Meanwhile, Pillet et al. [22] embarked on a complicated journey and developed a robust regression model using a comprehensively curated preoperative questionnaire. Their model relied on 57 comprehensive variables, culminating in the identification of four key predictors primarily related to DIE symptoms—pain duration, dysmenorrhoea, gastrointestinal discomfort and dyspareunia or subfertility. The commendable accuracy of the results is emphasised, although it should be recognised that the model is complicated and challenging for patients to use effectively. It is worth noting that this study was conducted in a specialist endometriosis centre, which may limit the generalisability of the results to non-specialist settings.

Perello et al. [23] contributed to the prediction of DIE on this topic with a retrospective study based on a dataset of women with histologically confirmed ovarian endometriomas. A number of variables were included in the model, including BMI (body mass index), age at baseline, history of surgery for endometriosis, and pain scores for dysmenorrhoea, dyschezia, dyspareunia and pelvic discomfort. Although the complexity of the model may be challenging for patients, its strength lies in its ability to predict the presence of ovarian endometriosis, potentially enabling prioritised treatment for affected patients.

## 5. Models for Predicting Endometriosis Location: Navigating the Landscape

In the field of endometriosis localisation prediction, two key studies shed light on this complicated terrain [19,20]. Fedele et al. [20] utilised the American Urologic Association Symptom Index (AUASI) questionnaire to develop a system for the pre-surgical diagnosis of endometriosis with bladder involvement. The effectiveness of the model was particularly evident in patients with suspected bladder involvement. Griffiths et al. [19] analysed the symptoms of their patients to detect patterns indicating an increased risk of rectovaginal endometriosis. Evidently, dyspareunia proved to be a common leading symptom in individuals struggling with this variant. The spectrum of symptoms included dysmenorrhoea, infertility, dyschezia, rectal pain, cyclical and non-cyclical rectal bleeding, diarrhoea and tenesmus. While these informative models are promising, they are tailored to populations who are symptomatic or already have endometriosis, limiting their applicability to the general population. Barcelos et al. [24] introduced a model for predicting the location of endometriosis by combining medical history, physical examination, parity, symptoms and imaging evaluation. The analysis of pre- and postoperative results facilitated the identification of variables relevant to the prognosis of endometriosis localisation. While this approach is impressively consistent with intraoperative findings in DIE cases, it requires imaging and is therefore not suitable as a stand-alone patient screening tool. Stegmann et al. [10] have developed a model to both predict the location of endometriosis and aid its identification during surgery. This dual-purpose model deciphered features that signify an increased or decreased likelihood of biopsy-confirmed endometriosis. While it provides valuable guidance for biopsy target selection, its dependence on additional elements emphasises that it requires additional validation. Chattot et al. [28] utilised a preoperative scoring paradigm to predict rectosigmoidal involvement in endometriosis patients. The remarkable efficacy of this study was slightly attenuated by the integration of magnetic resonance imaging (MRI) in conjunction with ultrasonography, which resulted in additional costs.

## 6. Predicting Models of Pregnancy after Endometriosis Surgery

In the field of pregnancy prediction after endometriosis surgery, significant progress has been made by Xin Li et al. [32], who validated the Endometriosis Fertility Index score (EFI) in women with historical endometriosis, up to 48 months after laparoscopy. This robust score combines medical history with surgical findings and paves the way for predicting the likelihood of spontaneous pregnancy. It is noteworthy that a direct correlation between increased EFI scores and an increased likelihood of spontaneous pregnancy was found. However, it should be borne in mind that the EFI does not take into account ovarian reserve and severe uterine anomalies or adenomyosis. In summary, if the EFI score is five or higher, natural conception should be considered after laparoscopic surgery to treat endometriosis, with in vitro fertilization (IVF) being a compelling alternative if natural conception is not possible.

## 7. Preoperative Predictive Model for Bowel Involvement in Cases of Endometriosis

Desplats et al. [25] investigated that rectosigmoidal endoscopic ultrasonography (RS-EUS) could serve as a predictive factor for patch or segmental resection in endometriosis when the rectosigmoidal nodule exceeds 5.20 mm. Although the results indicated a trend that wider nodes correlate with a higher likelihood of resection, statistical significance was lacking. Although RS-EUS has shown promise as a predictor, it remains secondary to other diagnostic tools in the diagnosis of endometriosis, warranting further investigation. Goncalves et al. [26] demonstrated the efficacy of transvaginal ultrasonography (TVUSS) with a sensitivity and specificity of 97% and 100%, respectively, in detecting rectosigmoid endometriosis nodules. However, when assessing intestinal mucosal infiltration, sensitivity and specificity fell to 62% and 83% respectively. Further studies investigated the diagnostic accuracy of 2D and 3D ultrasound [33], without finding a clear superiority between the two techniques. Bergamini et al. [27] investigated RS-EUS with transvaginal sonography with water contrast in the rectum (RWC-TVS) and found a higher sensitivity and specificity, although this was not statistically significant. Chattot et al. [28] introduced a preoperative scoring system integrating self-assessment questionnaires, speculum and digital examination, TVUSS and pelvic MRI to predict rectosigmoid involvement in endometriosis cases. Although the model was not validated and was performed at a single reference centre, it showed good results, albeit with a possible selection bias.

## 8. Discussion

Timely diagnosis of endometriosis is crucial to reduce patient frustration, fertility concerns and impaired quality of life [34]. This study aims to identify effective screening tools, with a focus on predictive models to assess endometriosis localisation and bowel involvement in deep infiltrating endometriosis (DIE). Focusing on these parameters is of central importance for early detection of the disease and crucial for planning optimal interventions [7]. Numerous question-based models have been explored, but their adoption remains hampered by inconclusive evidence [35]. Although the prediction of bladder or bowel disease shows positive trends [24], further evaluation is warranted. Geysenbergh et al. [36] addressed adolescents by adapting questionnaires for adults to identify potential endometriosis risk. However, the lack of similar studies for adults and the focus on urinary symptoms prevent wider application.

Predictive models play an important role in the context of endometriosis. They provide a strategic approach to understanding, diagnosing and managing this complicated and often debilitating condition. Their importance stems from several key factors in the field of endometriosis treatment. Firstly, these models enable early detection and diagnosis of endometriosis, even before extensive clinical signs appear. Early detection is crucial for timely intervention, which can lead to improved patient quality of life and more effective treatment outcomes. Secondly, predictive models are ushering in a new era of personalised medicine. By taking into account a range of variables, including symptoms, medical history and imaging results, these models enable customised patient care. This individualised approach makes it possible to tailor treatment plans to the unique characteristics of each case and predict outcomes with greater precision.

In addition, predictive modelling provides healthcare professionals with improved clinical decision support. This informed decision-making extends to treatment options, surgical planning and overall management strategies [16]. This in turn can optimise resource allocation and contain medical costs while improving patient outcomes. In addition, such models help optimise resource utilisation by identifying the patients who would benefit most from specialised procedures or interventions—a particularly important aspect given the limited resources available in healthcare. In addition, predictive models contribute to improved patient counselling by providing accurate information about the disease and possible outcomes. This enables patients to make informed decisions about their treatment and actively participate in the organisation of their healthcare. In addition, these models drive research and development efforts and shed light on the underlying mechanisms and risk factors of endometriosis. Another benefit is the reduction of diagnostic delays, as these models can identify patients at higher risk, leading to earlier intervention and treatment. The models also facilitate long-term monitoring of health status, help in the selection of participants in clinical trials and promote interdisciplinary collaboration between different medical disciplines [10].

While questionnaires offer valuable insights for patients [17], their implementation must take into account patients’ technological access. Predicting endometriosis using questionnaires remains a challenge when it comes to overcoming selection bias and population heterogeneity. To make accurate predictions, the biases in the prediction models need to be removed, especially when taking into account the different symptoms and localisations of the disease [37,38,39,40]. The diagnosis of endometriosis is associated with delays that affect patient well-being. Early detection allows patients to make informed decisions and facilitates physician engagement. The development of predictive models using machine learning methods, similar to cancer prediction, remains a goal. However, the complexity of endometriosis makes such modelling difficult given the heterogeneity of the disease and the variety of symptoms. In summary, symptom-based screening tools can help patients to recognise and understand their disease and at the same time help physicians to make decisions. However, the lack of a universally applicable and validated endometriosis screening tool compromises its clinical value. Future research should strive for a concise, accurate and widely applicable tool to improve the diagnosis and treatment of endometriosis. Limitations include heterogeneity of studies, different methodologies and retrospective designs. Further research must strive for consistency and validation to advance the field.

## 9. Conclusions

Endometriosis, a chronic disease that significantly affects women’s daily lives, benefits greatly from early diagnosis. Rapid identification enables optimal treatment, comprehensive risk awareness and fertility preservation. It is therefore essential to develop predictive models that enable doctors to recognize early symptoms with greater precision and thus provide better treatment. Unfortunately, the models proposed in the current literature often fall short of expectations and have unbalanced sensitivity and specificity. Moreover, they are usually complicated, lengthy and user-unfriendly. While the scientific community recognizes the need for prediction, the development of effective tools remains a challenge. It is important to develop a model in the near future that does not allow for subjectivity and complexity. It will be user friendly, will not take too long and will be validated on a large population. Artificial intelligence offers potential solutions, but large-scale, multicenter studies remain essential for substantial progress.

## Figures and Tables

**Table 1:** Databases search (PubMed and Embase).

Keywords	Number of Results
Endometriosis and predictive models	122
Fertility in Endometriosis and predictive models	39
Bowel operation for endometriosis and predictive models	19

**Table 2:** Inclusion and exclusion criteria.

Criteria	Inclusion	Exclusion
Population	Studies included women with symptoms of endometriosis	Studies where endometriosis is only confirmed by surgical intervention
Outcomes	Symptom based patients completed endometriosis questionnaires or predictive models. Evaluation of endometriosis with TV-USS or MRI	Tools other than patient-based questionnaires or predictive models
Study designs	Any type of study design	Editorials-Commentaries

**Table 3:** Characteristics of studies and measures.

References	Population and Country	Type of Tool	Brief Description	Clinical Utility	Validation and Performance
Abdulai et al. [7]	N = 12 Women with confirmed endometriosis willing to participate at the study Canada	Patient completed questionnaire and individually interview	Questionnaire used to ask participants to identify the information on endometriosis at website on endometriosis	Sex, Pain, and Endometriosis website and assess for destigmatizing properties of sexual health–related websites in general.	Not reported
Verket et al. [8]	N = 157 Women with surgically confirmed endometriosis Norway	Patient completed questionnaire	Questionnaire compared women with or without endometriosis depending on their answers	This model aims to identify women that potentially will develop endometriosis in the future	Validation needed externally Absenteeism is an important sign of possible endometriosis and early diagnosis in case of family history
Forman et al. [9]	N = 104 Women with Infertility more than 2 years UK	Patient completed questionnaire	Comparing sub fertile women with normal pelvis with endometriosis patients using a 7-point questionnaire	Questionnaire didn’t separate patients with or without endometriosis	Not reported
Stegmann et al. [10]	N = 119 Participants with history of CPP * underwent laparoscopy and excision of endometriotic lesion. Data added at training or test database UK	Data collection on endometriotic lesion’s characteristics	Women had laparoscopy and data were collected and added at a database	Model provides guidance on which areas of possible endometriosis should be biopsied.	Sensitivity 88.8% and specificity 24.6%. Validation is needed.
Fasciani et al. [11]	N = 120 Women referred with Chronic Pelvic pain or suspicion of endometriosis Italy	Endometriosis Index based on patient evaluation and diagnostic evidence	Use of 38 variables and parameters to predict endometriosis between patients with chronic pelvic pain and infertility	Positive outcome as screening tool but not clinical feasible as a patient completed measure	Score > 28 Prediction of DIE ** with 72.4% sensitivity and 90.1% specificity
Yeung et al. [12]	N = 90 Women referred with Chronic Pelvic pain or suspicion of endometriosis United States	Predictive mathematical model for early-stage endometriosis	Preoperative questionnaire based on demographics and patients’ medical history, predictive model included 5 factors	Allows the estimation of an individual probability, but not clinical feasible as a patient completed measure	High discrimination ability Sensitivity 80.5% Specificity 57.7%
Eskenazi et al. [13]	N = 90 Women undergoing laparoscopy or laparotomy (study sample) N = 120 Women underwent laparoscopy (test sample) Italy	Patient interviews and noninvasive diagnostic procedures	Patients’ interview to predict surgical diagnosis of endometriosis	Ultrasound positive at 100% in ovarian endometriomas but failed to diagnose non ovarian endometriosis Pelvic examination 100% in ovarian endometriomas, 44% in non ovarian endometriosis	66% of cases with correct classification Validation is not reported
Calhaz-Jorge et al. [14]	N = 1079 Subfertile women undergoing diagnostic or therapeutic laparoscopy Portugal	Predictive mathematical model	Interviewer collected data on medical history and demographics to evaluate whether they can predict endometriosis	Dysmenorrhea had a high predictive value, but not the same for dyspareunia. Not clinical feasible as a patient completed measure	Multivariate prediction model had an area under the ROC curve of 0.71 for all endometriosis and 0.74 for grade II/IV Validation not reported
Ballard et al. [15]	N = 185 Laparoscopy for CPP * UK	Patient completed questionnaire	40 pain descriptors for 3 different aspects of pain: descriptions of pain, intensity, anatomical areas of pain	Throbbing pain and dyschezia can be helpful for differentiate diagnosis of women with or without endometriosis	Performance not reported
Hackethal et al. [16]	N = 69 Women with suspected or known endometriosis Germany	Patient completed questionnaire	Prospective and preoperative questionnaire on medical history and pain characteristics	Questionnaire did not aim to differentiate women with or without endometriosis. Not clinically feasible as too long	Performance not reported
Nnoaham et al. [17]	N = 1396 Diagnostic laparoscopy performed at women with dysmenorrhea, dyspareunia, non-menstrual pelvic pain, menstrual dyschezia or infertility 13 countries	Predictive symptom-based model	Variables used to investigate if they can predict the presence or not of endometriosis in laparoscopy	Models showed to be poor in prediction of endometriosis, accuracy higher when there is evidence of ovarian cysts or nodules at ultrasound. Endometriosis III/IV stage predicted in higher accuracy	Area under ROC curve = 0.683 The extent of models’ predictive power in self-selected women with pelvic pain is unknown
Endometriosis self test [18]	United States	Patient completed questionaire	Self scoring (yes or no), that could lead women to understand if they have endometriosis and seek guidance	3 or more positive women are considered positive for endometriosis	Performance not reported
**Site specific endometriosis studies**					
Griffiths et al. [19]	N = 51 Women referred for endometriosis and undergoing laparoscopy UK	Observational retrospective analysis of patient reported symptoms	To calculate the risk of rectovaginal endometriosis depending on symptoms	Utility of detecting endometriosis at general population is low	Nausea, abdominal bloating were strong markers for rectovaginal disease with a predictive prevalence of 87 and 89% respectively
Fedele et al. [20]	N = 157 Women undergoing laparoscopy or laparotomy for CPP * Italy	Partial modification of the amreican Urologic Association Symptom Index (AUASI)	Presurgical diagnosis of bladder endometriosis using a questionnaire	Utility of detecting endometriosis at general population is low	High accuracy for population that bladder endometriosis is expected. Area under ROC curve was 0.951
DIE ** studies					
Chapron et al. [21]	N = 134 Women schedule for laparoscopy for CPP * France	Diagnostic model based on symptoms that are collected via a self-administered questionnaire	Prediciting posterior DIE when wonen are presented with dysmenorrhea, dyspareunia, nonmenstrual pain, urinary and gastrointestinal symptoms during period	Strongest prediction factor for posterior DIE ** is dyschezia. Posterior DIE ** is not related with only. dyspareunia	Area under the ROC was 0.77 sensitivity was 74.5%, specificity 68.7%.
Lafay Pillet et al. [22]	N = 326 Women with histological confirmation of endometriosis having complete treatment of endometriotic lesions France	DIE score calculated from a regression model, from a preoperative symptom questionnaire	Diagnostic score based on 57 variables	Diagnostic tool with good diagnostic performance.	With score 35 or more probability of DIE ** = 88%, 94% specificity. Score 13 and less probability of DIE ** = 10%, 95% specificity.
Perello et al. [23]	N = 178 Women with ovarian endometriosis, removal of endometriosis Spain	Retrospective analysis of women with endometrioma undergoing surgery	Prediction model for ovarian endometriomas	Model showed discrimination in predicting development of DIE ** in patients with ovarian endometriomas	Area under the ROC curve was 0.91, with sensitivity 80% and specificity 84%
Barcellos et al. [24]	N = 46 Women undergoing surgery for DIE ** Brazil	Assessment of clinical and anatomic sites using Lasmar map	Assessment of clinical and anatomic sites depending on medical history	Diagnostic approach including imaging evaluation rather than symptoms only	Lasmar map has high sensitivity, specificity and accuracy in recognizing the main site of endometriosis without laparoscopy
**Studies on Prediction models for bowel resection**					
Desplats et al. [25]	N = 73 To assess whether the lesion features observed via preoperative rectosigmoid endoscopic ultrasonography (RS-EUS)might predict the need for bowel resection. France	Retrospective analysis on patients underwent resection of nodule of endometriosis, by selecting the pre-operative data of RS-EUS	When a rectosigmoid nodule is >5.2 mm thick on RS-EUS, this can predict the need for resection	Use RS-EUS at pre-operative assessment of women with endometriosis	Sensitivity 76% and specificity 81% Validation is needed.
Goncalves et al. [26]	N = 194 The objective of this study was to determine the capability of TVUS-BP **** to predict the presence of one or more rectosigmoid nodules and the deepest bowel layer affected by the disease. Brazil	A prospective study with clinical and TVUS-BP **** suspected deep endometriosis submitted to videolaparoscopy. Image data were compared with surgical and histological results.	TVUS-BP **** performed preoperatively and then results compared with histology post-operatively	TVUS-BP **** is an adequate exam for evaluating the presence of one or more rectosigmoid nodules and the deepest layer affected in deep infiltrating bowel endometriosis, confirming the importance of this technique for defining the most appropriate surgical strategy to be implemented.	With respect to bowel nodule detection and presence of at least two rectosigmoid lesions, TVUS-BP had a sensitivity of 97 and 81%, specificity 100 and 99%, positive predictive value (PPV) 100 and 93% and negative predictive value (NPV) 98 and 96%, respectively. Regarding diagnosis of infiltration of the submucosal/mucosal layer, TVUS-BP had a sensitivity of 83%, specificity 94%, PPV 77%, NPV 96%.
Bergamini et al. [27]	N = 61 To evaluate the accuracy of Transrectal Sonography (TRS *****) and a new technique, Transvaginal Sonography with Water-Contrast in the Rectum (RWC ******-TVS), in the diagnosis of rectosigmoid endometriosis Italy	Patients who underwent laparoscopy or laparotomy for suspected rectosigmoid endometriosis.	The accuracy of RWC ******-TVS in the detection of intestinal stenosis was evaluated comparing the radiologic and ultrasonographic results with the macroscopic findings at surgery and pathology.	RWC ******-TVS diagnosed rectosigmoid endometriosis with the same accuracy of TRS *****. RWC ******-TVS is a new, simple technique for a single-step and accurate preoperative assessment of rectosigmoid endometriosis.	For the diagnosis of rectosigmoid endometriosis the sensitivity, specificity, positive and negative predictive values of TRS ***** and RWC ******-TVS were 88.2% and 96%, 80%, and 90%, 95.7%, and 98%, and 57.1% and 81.8%, respectively
Chattot et al. [28]	N = 119 Study aims to create and validate a pre-operative score to predict rectosigmoid endometriosis France	Data collected from self-assessment questionnaire, digital and speculum examination, transvaginal ultrasound and MRI	Four variables: palpation of posterior nodule on digital examination, ultrasound findings, rectosigmoid infiltration on MRI, blood in stools during menstruation	Score was derived at high risk and intermediate group	High risk sensitivity of 100% for RE ***, intermediate had probability of 42%. Validation externally is needed

* CPP: Chronic Pelvic Pain. ** DIE: Deep Infiltrated Endometriosis. *** RE: Rectosigmoid endometriosis. **** TVUS-BP: Transvaginal ultrasonography with bowel preparation. ***** TRS: Transrectal Sonography. ****** RWC-TVS: Transvaginal Sonography with Water-Contrast in the Rectum.

## Data Availability

All data are presented in the present manuscript.
